# Accept or Refuse? A Pilot Study of Patients' Perspective on Participating as Imaginary Research Subjects in Schizophrenia

**DOI:** 10.4306/pi.2009.6.2.66

**Published:** 2009-06-30

**Authors:** Jin Hun Kim, Daeho Kim, Sung-Hyouk Park, Junghyun Nam

**Affiliations:** 1 Neuropsychiatry Research Laboratory, Gongju National Hospital, Gongju, Korea.; 2 Department of Psychiatry, College of Medicine, Hanyang University, Seoul, Korea.

**Keywords:** Informed consent, Patient participation, Demography, Schizophrenia, Clinical research

## Abstract

**Objective:**

The goal of the present study was to evaluate demographic and clinical factors that affect the intention to participate in commonly-conducted research in patients with schizophrenia.

**Methods:**

Thirty-four outpatients with a diagnosis of schizophrenia were enrolled in this study. They were asked whether they would have any intention to participate in four imaginary studies: a simple questionnaire, a genetic study, a study of complex tasks and a risky study. We analyzed the differences in general psychopathology, insight and demographic characteristics of the participants according to their responses (acceptance or refusal) to the four proposed studies.

**Results:**

Younger and better-educated patients tended to decline participation in a risky study. Patients with a longer duration of regular psychiatric follow-ups tended to willingly participate in the simple questionnaire. There were no overall statistical differences in general psychopathology and insight between patients who agreed or declined to participate in studies.

**Conclusion:**

Age and education level may be factors that influence decisions to participate in schizophrenia studies. Further research is needed to confirm and expand on the current findings.

## Introduction

Voluntarism is the most basic ethical issue in medical research and human rights.^1^ Debate of the ethical issues surrounding medical research began mainly after the Nazis performed non-consensual experiments on individuals under arrest. These debates resulted in the declaration of ten principles to govern studies using human subjects. Named after the important trial of Nazi war crimes, the Nuremberg Code was established as the most famous code of ethics relating to medical research.^2^ Thereafter, voluntary participation became an essential part of conducting research with human subjects. Recent developments in psychiatric research have introduced methodology varying in complexity, from simple questionnaires to neuropsychological study, and from genetic studies to new drug development trials. Consideration given to subject recruitment has become more complex and detailed.^3^-^5^ Informed consent can be defined as the process by which individuals make free decisions with adequate knowledge about the consequences of participation. Informed consent has been particularly important in psychiatric research, owing to the debate over the ability of patients to provide it. Specific conditions of the patients make this a more relevant topic in psychiatry compared with other medical fields.^6^-^8^ Disabilities due to psychiatric symptoms are likely to interfere with appropriate methods of informed consent.^9^ The minimal components that fulfill an appropriate informed consent process are understanding, intention and voluntariness.^10^ Some have also added communication skills to the components described above.^11^ Understanding implies the ability to comprehend the purpose, procedure, risk and benefit of a study. Intention is the willingness, which reflects individual values, to participate in a study after consideration of the suggestion by a researcher or a treating psychiatrist. Voluntariness is related to intention, implying that the person's decision is free of outer or inner enforcement.^12^ Unfortunately, some psychiatric patients are unable to fulfill the above conditions.^13^-^23^

Data about factors related to influence on, or the capacity to provide, informed consent, have accumulated. However, the influence of patients' demographic or clinical characteristics on their perspective on study participation has not been evaluated fully. For example, if the patients' perspectives on the study resulted in the exclusion of a specific population, selection bias could limit the generalizability of the study.^24^-^26^ This problem is particularly difficult because it cannot be corrected or modified after study completion.^27^,^28^ The difference in the psychopathology and in detailed clinical variables could not be obtained from individuals who decline to participate in the study, as they would not provide a detailed clinical history. Questionnaires about imaginary studies can be an alternative to studying this topic, and can give us a more comprehensive view of selection bias and the factors related to intentions of specific study subjects. Moreover, the information could help to determine which studies may be acceptable while others are refused. However, little data is available regarding the factors related to patients' participation in specific studies.

The ability to evaluate the intention of schizophrenic patients can reveal useful information about the selection bias on patients' perspective on being a part of three common types of procedures for which informed consent is required. The purpose of this exploratory study was to examine how psychopathology and other patient variables could be related to the likelihood that they would be inclined to participate in four imaginary studies designed to reflect actual clinical practices.

## Methods

The study population consisted of 34 subjects who gave informed consent to participate in this study. The subjects were recruited from the Seoul National Hospital, Seoul, Korea. All participants were outpatients diagnosed with DSM-IV-TR^29^ Schizophrenia. Diagnosis was confirmed by the agreement of two psychiatrists. Clinical and demographic characteristics such as sex, age, education level, employment status, previous history of study participation, and duration of continuous contact with the primary care psychiatrist were collected. Based on patients' answers, their history of previous study participation was divided into three groups: simple questionnaire study, open-label drug study and double-blind placebo study. The Positive and Negative Syndrome Scale for Schizophrenia (PANSS)^30^ was used to evaluate their psychopathology and the General psychopathology item 12 (G12) of PANSS (lack of judgment and insight)^30^ was used to assess the level of insight of the patients.

### Study tools: informed consent to imaginary studies

We developed questionnaires to evaluate the intention to participate in four imaginary studies. We named these questionnaires the simple questionnaire study (SS), the genetic study (GS), the complex study (CS) and the risky study (RS), according to the main feature of each study. The procedure of the current study was very simple. We presented the patients with informed consent forms for the four imaginary studies, and asked them whether they would participate in these studies if they were real. In addition to the basic explanation of each study, the following emphases were used. SS was a simple pen and paper questionnaire study, GS required blood sampling, CS required a complex neuropsychological battery and some time to complete and RS required them to switch their current antipsychotic medication and undergo a double-blind procedure, if needed. Table 1 summarizes the main point of each study explanation. Other procedures followed a typical study design in each study area. Possible adverse events and gains were also explained. We minimized selection bias due to misunderstanding of each study purpose by using a figure and flow chart method, since the understanding or misunderstanding of a specific study is of interest to the current study.

### Statistical analysis

Spearman's correlation coefficient was used to evaluate the correlation between the history of previous study participation and the four imaginary study participation intentions. Patients' clinical variables and demographic characteristics were compared using either independent t-test or Fisher's exact test between study participants and study refusal subjects in 4 imaginary studies. All statistical analysis was performed using the Statistical Package for the Social Sciences (SPSS) 15.0 Windows version, and the significance level was set at p<0.05.

## Results

### Clinical variable and demographic characteristics

Study subjects comprised slightly more men than women (n=18, 52.90%) and the mean age was 37.26 years. The simple questionnaire study was the most common procedure previously experienced by the subjects (n=24, 70.60%). The mean duration of illness was 11.91 years. The mean duration of continuous contact with a primary care psychiatrist was 20.71 months. The mean PANSS score was 68.09 and the mean G12 score of PANSS (higher scores mean greater lack of insight)^30^ was 3.50. The clinical variables and demographic characteristics are illustrated in Table 2.

### Patients' willingness to participate overall and in each study

**FIGURE 1:**
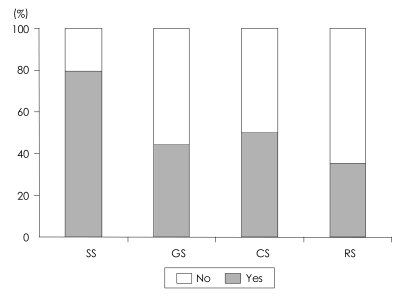
Percentage of patients agreeing to participate in each type of study. SS: simple study, GS: genetic study, CS: complex study, RS: risky study, No: unwilling to participate in the study, Yes: willing to participate in the study.

We obtained a total of 54 informed consents, out of 136 possible study participations (34 people×4 questionnaires). Thus, the study population agreed to participate at the rate of 39.71%. As shown in Figure 1, subjects were often willing to participate in SS, they were less likely to participate in GS. The intentions of participations between the GS, CS, and RS were significantly correlated with each others, but not the SS (Table 3).

### The relationship between previous history of study participation and current study participation

As shown in Table 4, the subjects' previous history of study participation was not correlated with their participation in the current imaginary studies.

### The relationships between demographic or clinical variables and study participation intentions

In RS, there was significant difference in the age and years of education between study participants and non-participants. In SS, study participants had a significantly longer duration of continuous contact with a primary care psychiatrist compared with study non-participants. There were no differences in PANSS and G12 of PANSS between study participants and study non-participants in all four studies. These results are summarized in Table 5.

## Discussion

We investigated the intention of schizophrenia patients to participate in four imaginary studies developed by us. We explored the factors related to their intention to participate in each study.

In this study, about 40% of all study subject patients chose to participate in at least one imaginary study. A relatively low proportion of patients were willing to participate in GS or RS. This result showed that patients were considering possible risks related to a specific study. Blood sampling and placebo clinical trials pose a potential risk for patients who participate. The current study population consisted of relatively clinically-stable outpatients who had little desire to expose themselves to a specific risk. As a result, GS or RS studies likely attract only a specific subset of the entire schizophrenic population. However, this possible selection bias occurs only in the case of SS and RS. Younger and more highly-educated people tended to decline participation in the RS study. This may reflect the greater potential for risk evaluation, although the current study did not explain the phenomenon fully. For SS, a longer duration of contact with a primary care psychiatrist was correlated with willingness to participate in a SS. Rapport or compliance can affect the intention of research participation in SS. Yet, only seven patients chose non-participation; such a small sample size means the result cannot be explained fully in the current context and the conclusion must be deferred. Another interesting result of this study was the correlation between the patients' intention to participate in each study. These correlations were found in all imaginary studies except GS. As described above, the lack of willingness to participate in GS could be related to another possible factor. However, participation in one study can be correlated with participation in another study. As one patient's perspective revealed, the three (GS, CS, RS) studies were viewed in a similar way and a similar mechanism was used to decide willingness of study participation. Willingness of study participation was very similar across different study subjects in people with schizophrenia; other clinical variables and demographic characteristics were not different between those who chose or declined to participate. Taken together, the current findings indicate that a specific population of schizophrenia patients is likely to participate or decline to participate in research, regardless of the study type. However, the choice to participate in a specific study appears to relate not to the severity of the patients' psychopathology, but rather to demographic factors (i.e., age and level of education).

Our exploratory study had several limitations. The study sample was small and exclusive to schizophrenia outpatients, which limits the ability to generalize the results of this study to the general schizophrenia population. The lack of significant results for some variables, could have been due to low statistical power. In contrast, multiple analyses could have result in type I errors since we analyzed the difference of some demographic data without accurate study hypothesis or interest of variable. In this study, we included only some clinical and demographic variables; neuropsychological tests or comprehensive clinical variables would strengthen the predictive power of our approach, and could give us greater insight into other factors related to the intention of study participation. Another limitation is the inability to compare current study results to previous results, as few results from previous literature are relevant to the approach taken here. It is possible that there would be some difference in subjects' responses when faced with real as opposed to imaginary choices. In addition, the validity of the research questionnaire was not assessed, since this was outside of the scope of this pilot study. Despite these limitations, the results of this pilot study, in conjunction with other studies on the subject, provide a point of reference for the field of schizophrenia research.

In conclusion, some demographic or clinical factors could be related to a specific study type, and patients' participation in each of the different studies is correlated. Further studies are needed to clarify this issue and contribute to an improved framework for interpreting research results in schizophrenia research.

## Figures and Tables

**TABLE 1:**
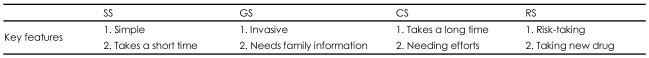
Key characteristics of the four current study types

SS: simple study, GS: genetic study, CS: complex study, RS: risky study

**TABLE 2:**
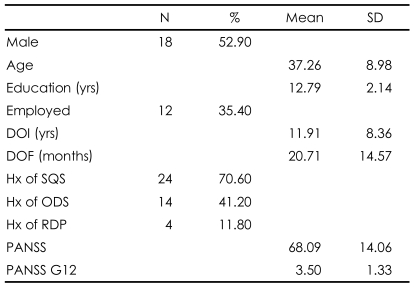
Baseline characteristics of participants (N=34)

SD: Standard deviation, DOI: duration of schizophrenia illness, DOF: duration of follow-up with the primary care psychiatrist in outpatient department, Hx: history, SQS: simple questionanaire study, ODS: open-label drug study, RDP: randomized double blind placebo controlled sutyd, PANSS: Positive and Negative Syndrome Scale for Schizophrenia, G12: General psychopathology item 12

**TABLE 3:**
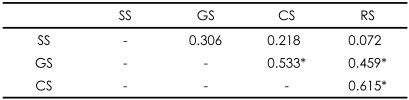
Spearman's Correlation Coefficient between patients' willingness to participate in the four current study types (N=34)

^*^p<0.05. SS: simple study, GS: genetic study, CS: complex study, RS: risky study

**TABLE 4:**
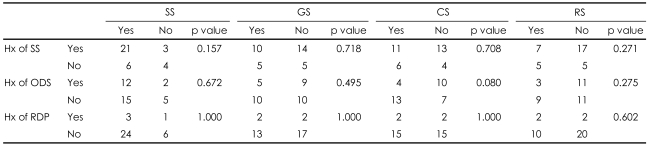
Relationship between previous history of study participation and the willingness to participate in the current four study types. Data were calculated by Fisher's exact test (N=34)

SS: simple study, GS: genetic study, CS: complex study, RS: risky study, Hx: history, SS: simple questionnaire study, ODS: open-label drug study, RDP: randomized double-blind placebo-controlled study

**TABLE 5:**
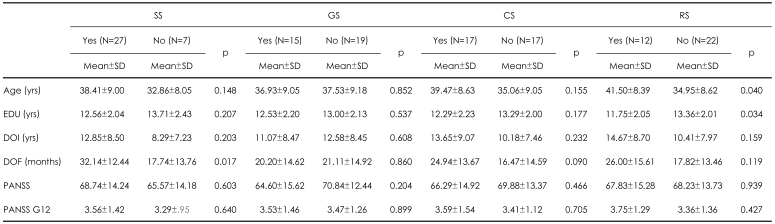
Differences in demographic and clinical variables between those willing or unwilling to participate in the 4 current study types (N=34)

SS: simple study, GS: genetic study, CS: complex study, RS: risky study, EDU: education, DOI: duration of schizophrenia illness, DOF: duration of follow-up with the current research doctor in outpatient department, Hx: history, SS: simple questionanaire study, ODS: open-label drug study, RDP: randomized double-blind placebo-controlled study, PANSS: Positive and Negative Syndrome Scale for Schizophrenia, G12: General psychopathology item 12, YES: willing to participate in the study, NO: unwilling to participate in the study
